# Acute pituitary crisis after lumbar surgery: A case report

**DOI:** 10.1097/MD.0000000000036294

**Published:** 2023-12-01

**Authors:** Hui Wang, Xingrui Gong

**Affiliations:** a Department of Anesthesiology, Xiangyang Central Hospital, Affiliated Hospital of Hubei University of Arts and Science, Xiangyang, Hubei, China.

**Keywords:** anesthesia, case report, hypopituitarism, Sheehan syndrome

## Abstract

**Rationale:**

Patients with hypopituitarism often have no specific symptoms; that frequently results in missed diagnosis. An acute hypopituitarism crisis can be induced under stressful conditions. Here, we report a rare case of an acute pituitary crisis after lumbar surgery.

**Patient concerns:**

We describe a 62-year-old female who presented with refractory electrolyte disorders after lumbar surgery. In addition, she developed anorexia, nausea, vomiting, chest cavity effusion, ascites, pericardial effusion, anemia, low blood pressure, bradycardia, and unconsciousness after surgery. MRI showed an empty sella turcica.

**Diagnoses:**

She was diagnosed with postoperative acute hypopituitary crisis.

**Interventions:**

The patient received hormone replacement therapy.

**Outcomes:**

Her symptoms improved significantly following the initiation of hormone replacement therapy and was well 6 months after surgery.

**Lessons:**

Refractory postoperative complications, including electrolyte disorders, infection, nausea, vomiting, circulatory collapse, anemia, and coma, indicate an acute postoperative hypopituitary crisis.

## 1. Introduction

Hypopituitarism is characterized by a decrease in or absence of one or more hormones secreted by the pituitary gland. This disease is mostly caused by necrosis or tumors of the pituitary gland. The symptoms of hypopituitarism vary from selective hypopituitarism to panhypopituitarism.^[1]^ Acute hypopituitarism rarely occurs, and the majority of patients present with nonspecific symptoms in the chronic phase, including fatigue, cold intolerance, sparse axillary and pubic hair, hypopigmentation, and dry skin.^[2]^ Acute pituitary crisis is mostly caused by infection, trauma, tumors, or surgery. Here, we present a case in which a female developed refractory electrolyte disorders, circulatory collapse, anemia, and coma after lumbar surgery. She was later diagnosed with an acute pituitary crisis. The patient was discharged after receiving hormone replacement therapy. Early suspicion of hypopituitarism is important for the prevention of postoperative complications. To better recognize and remember perioperative complications, we described the multiple presentations after surgery and reviewed some anatomical, vascular, and physiological aspects of the pituitary glands. We hope that our case will help diagnose hypopituitarism-related complications perioperatively.

## 2. Case report

A 62-year-old female patient was admitted to the Xiangyang Central Hospital September 23, 2020 due to lumbar pain and weakness in both lower extremities for ten years. Physical examination showed low back pain with increasing press. Magnetic resonance imaging (MRI) showed lumbar 4/5 spondylolisthesis using. Laboratory tests showed a hemoglobin level of 93 g/L, and other laboratory tests were normal. The patient had a history of sparse menstrual cycle after delivery, ceased menstrual cycle at the age of 45 years. In the past 3 years, the patient developed progressive fatigue, anorexia, cold intolerance, sparse axillary and pubic hair, and was unwell. She did not receive any medical interventions previously. Standard care is performed, so ethical approval is not applicable. Written informed consent was exempted from the patient.

The patient was diagnosed with lumbar 4/5 spondylolisthesis and underwent spinal canal decompression and internal fixation surgery under general anesthesia. The surgery lasted 6 hours and had 800 mL of blood loss and a total of 2000 mL of Ringer solution, 6 U concentrated red blood cells and 400 mL of plasma were infused intraoperatively. Refractory hypotension was found and intraoperative hemodynamics were maintained with noradrenaline. The patient recovered and was extubated 30 minutes after the end of the surgery and returned to the ward after an hour of postoperative intensive care unit stay. There was no obvious abnormality at the 72-hour follow-up after the operation.

On postoperative days 1,3, and 5, the patient received 2, 1.5, and 1.5U concentrated red blood cells and 400 mL of plasma on postoperative day 5 to remedy anemia. On postoperative day 8, the patient developed lethargy, blurred vision, and unconsciousness. An acute blood gas analysis showed that the sodium was 115.3 mmol/L; thus, concentrated sodium was infused immediately, and the patient recovered rapidly. In addition, until postoperative day 10, the patient incision continued to exudate fluid. Considering the subcutaneous infection, emergency surgery was initiated to remove effusions beneath the incision under general anesthesia on postoperative day 11. The patient recovered and was returned to the ward, followed by disinfection therapy.

On postoperative day 16, the patient complained of abdominal distension, anorexia, and vomiting, followed by cough, chest tightness, and dyspnea. Quickly, the patient developed a low blood pressure (70/40 mm Hg) and low oxygen saturation (88%). The laboratory test results showed as follows: sodium,130 mmol/L, hemoglobin, 97 g/L, brain natriuretic peptide, 24466.8pg/mL, TP, 53 g/L. Doppler ultrasound revealed bilateral chest cavity effusion, ascites, and pericardial effusion. The patient received potassium, concentrated sodium, and closed thoracic drainage immediately, and the symptoms improved quickly after treatment.

**Figure 1.**
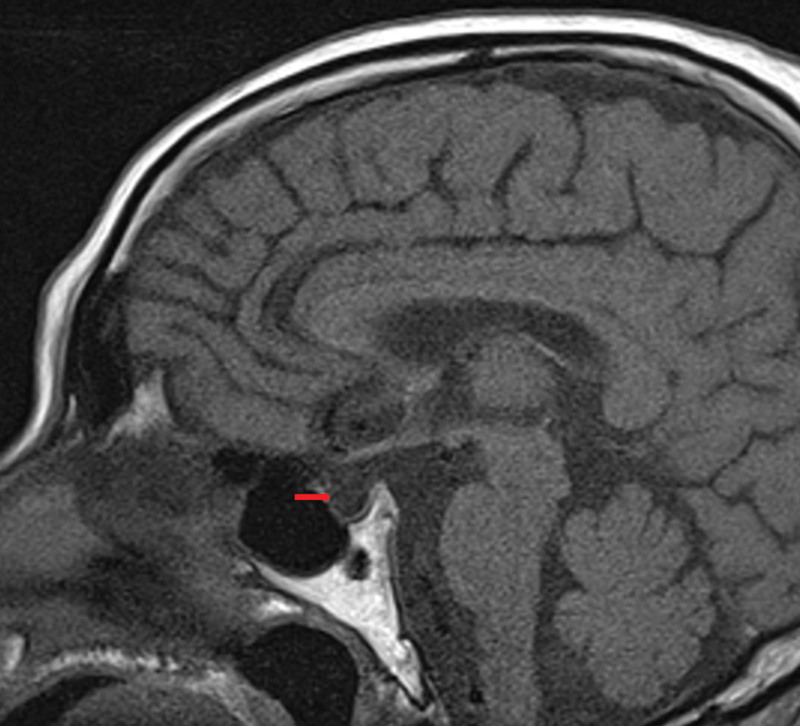
The empty sella turcica in the MR (Red arrow).

On postoperative day 18, the patient presented with repeated episodes of unconsciousness accompanied by involuntary convulsions in both upper limbs. Electrocardiography showed bradycardia, premature ventricular contractions, blood potassium is 2.8 mmol/L and blood sugar is 3.3 mmol/L. glucose and potassium were infused intravenously and low blood sugar and potassium levels were corrected quickly. A multidisciplinary consultation was initiated at the same time, and a hypopituitarism crisis was considered. The laboratory results showed T3: 0.52 nmol/L, free T3, 1.97pmol/L, T4, 18.82 nmol/L, free T4, 2.76 pmol/L, TSH, 4.50 mIU/mL, TPO-Ab, 63.18 IU/mL, Cortisol, 2.19 ug/dl, ACTH, 17 pg/mL, Pituitary Hormone: FSH, 8.70 IU/mL, LH, 5.02 IU/mL, PRL, 1.44 ug/L. Pituitary MR was considered and the results showed an empty sella turcica (pituitary atrophy, Fig 1).

The patient was diagnosed with postoperative acute hypopituitary crisis. Hydrocortisone, prednisone, and levothyroxine were indicated for hormone replacement therapy. All of the symptoms improved quickly after the treatment commencement and the patient was discharged a week later. Six months after surgery, the patient felt well and had no such symptoms related to hypopituitary symptoms. The patient was very satisfied with the treatment.

## 3. Discussion

This case highlights that a missed diagnosis of perioperative hypopituitarism is a risk factor for postoperative complications. Various refractory postoperative complications, especially electrolyte disorders, anemia, and recurrent unconsciousness, suggest a suspicion of pituitary crisis.

Hyponatremia is the most commonly observed electrolyte disorder in hypopituitarism.^[3]^ Low blood pressure and volume stimulate the release of antidiuretic hormones. Hypothyroidism and glucocorticoid deficiency result in decreased free water clearance and subsequent hyponatremia independent of antidiuretic hormones. Severe electrolyte disorders can result in arrhythmia or even unconsciousness. In our case, the patient presented with refractory hyponatremia, which was not resolved before pituitary hormone replacement therapy. Hypoglycemia is another common complication of hypopituitarism owing to its low cortisol content.^[4]^ The patient in our study developed recurrent unconsciousness probably secondary to hypoglycemia and hyponatremia. The potassium concentration was normal most of the time, as adrenal production of aldosterone is independent of the pituitary function. The hypokalemia reported on postoperative day 18 could be due to gastrointestinal loss after nausea, vomiting, and diarrhea. Other electrolyte abnormalities, including hypokalemia, hypomagnesemia, hypocalcemia, and hypophosphatemia may happen in the setting of hypopituitarism.^[3]^

Some patients with hypopituitarism may also show anemia, thrombocytopenia, pancytopenia, and coagulation disorders.^[5]^ Cortisol and thyroid hormone deficiencies are involved in the development of anemia by lowering erythropoietin synthesis of erythropoietin.^[6]^ The patient in our case had respiratory and circulatory failure symptoms, which were attributed to bilateral chest cavity effusion, ascites, and pericardial tamponade, which was confirmed by ultrasound B. The cause of multiple-cavity effusion could be due to a deficiency of hypothyroidism and low albumin levels. Early recognition of pericardial and chest effusion and its underlying causes is important for improving prognosis and avoiding unnecessary invasive investigations. Hyperlipidemia^[7]^ and psychiatric and neurocognitive impairment^[8]^ are common complications of hypopituitarism; however, in our study, the patient did not present with these symptoms.

The general principle in the treatment of patients with hypopituitarism is hormone replacement.^[9]^ We first used hydrocortisone and methylprednisolone, followed by levothyroxine. Early thyroxin therapy can exacerbate glucocorticoid deficiency and induce adrenal crisis by increasing whole-body metabolism. Hormone replacement therapy does not improve pituitary function or prevent the progression of pituitary necrosis. However, it rapidly ameliorates the symptoms and signs of hypopituitarism. The patient felt well and was discharged rapidly after hormone replacement treatment.

When referred to the previous history, the patient reported a sparse menstrual cycle for many years and progressive fatigue, anorexia, cold intolerance, sparse axillary and pubic hair, and was unwell for 3 years. The patients may have hypopituitarism and were not diagnosed for a long term. Stress factors such as infection, surgery, trauma, and tumors trigger an acute hypopituitary crisis. Our patient presented with a postoperative hypopituitarism crisis may be attributed to surgical stress and bleeding. Sheehan syndrome after delivery is a complication of postoperative hemorrhage and is the most common cause of hypopituitarism. As the patient reported postpartum hemorrhage 30 years ago and the characteristics of the slow progression of the disease, we have reason to speculate that the hypopituitarism may have been attributed to the Sheehan syndrome 30 years ago.

Sheehan syndrome is a rare complication that occurs in 5 of every 100 million pregnancies and is the most common cause of hypopituitarism in developing countries.^[10]^ During pregnancy, the pituitary gland increases by 45% during the first trimester and reaches up to 2 to 3 of its original size and its highest volume during the first few weeks of the postpartum period.^[11]^ The enlargement of the pituitary gland during pregnancy is explained by hyperplasia of prolactin-secreting (lactotroph) cells in the anterior pituitary gland.^[12]^ An increased size of the pituitary gland requires more blood supply, while the vasculature is compressed by sella turcica, which results in pituitary ischemia during pregnancy. Postpartum hemorrhage exacerbates low perfusion of the pituitary gland and results in Sheehan syndrome.

The acute presentation of Sheehan syndrome immediately after an eventful delivery makes diagnosis easy. Acute Sheehan syndrome presents with sudden onset of severe headache, loss of vision, and unconsciousness.^[13]^ whereas mild pituitary gland injury may induce an auto immune response and the patient could go asymptomatic for many years. Structural changes show an enlarged non-hemorrhagic pituitary gland with central infarction; then, the gland shrinks and progresses by atrophy over months to years, and finally, an image of an empty sella turcica.^[14]^ Empty sella turcica can be considered a characteristic of Sheehan syndrome diagnosis. In our case, the patient had an empty sella turcica; however, this was not observed until the pituitary crisis was suspected. Patients may present with partial or panhypopituitarism, depending on the degree and site of necrosis. If more than 70% of the anterior pituitary gland is affected, patients present with symptoms and signs.^[15]^ A history of lactation failure and absence of resumption of normal menstrual cycles after delivery raise suspicion of Sheehan syndrome,^[16]^ while the absence of these symptoms does not exclude Sheehan syndrome, as some patients may be asymptomatic for a long time.^[1]^

In most cases, patients with Sheehan syndrome have a slow progression of pituitary dysfunction, even many years after the initial insult.^[17]^ Because of the chronic progression of Sheehan syndrome, most patients (>50%) present with nonspecific symptoms years after delivery. The delay between diagnosis and a previous pituitary insult is reported to be 7 to 19 years^[14]^ for incomplete hypophyseal damage after delivery. Studies have identified the autoimmune profile of the pituitary gland and hypothalamus, and lymphocyte cells were found in the gland.^[18]^ Sheehan syndrome frequently presents with fatigue, dry skin, increased wrinkling around the mouth and eyes, hypopigmentation, sparse axillary and pubic hair, slowing of reflexes, bradycardia, or sometimes coma in the chronic phase.^[19]^ In our case, the patient had some nonspecific symptoms, that did not catch the doctor attention and resulted in a missed diagnosis before surgery.

Our study has limitations that the preoperative hypopituitarism was diagnosed by previous postpartum hemorrhage and subsequent nonspecific symptoms rather than laboratory test. The extent to which the pituitary hormones deficiency preoperatively could be evaluated.

In conclusion, we reported a very rare case of missed diagnosis of asymptomatic and slow-progressing hypopituitarism. Preoperative undiagnosed hypopituitarism increases perioperative morbidity and mortality rates. Sparse menstrual cycle or premature menopause, progressive fatigue, anorexia, cold intolerance, sparse axillary and pubic hair, and unspecific symptoms highly suggest a latent preoperative hypopituitarism. Whereas refractory postoperative electrolyte disorders, anemia, nausea and vomiting, circulatory collapse, and unconsciousness indicate a postoperative hypopituitary crisis. Evaluation of a history of postpartum hemorrhage, pituitary MRI, and laboratory hormone tests aids in the diagnosis and perioperative management of an acute hypopituitary crisis.

## Author contributions

**Data curation:** Hui Wang.

**Supervision:** Xingrui Gong.

**Writing – original draft:** Xingrui Gong.

**Writing – review & editing:** Xingrui Gong.

## References

[1] Kovacs K. Sheehan syndrome. Lancet. 2003;361:520–2. 12583962 10.1016/S0140-6736(03)12490-7

[2] Thyagaraj V Kumar MJ. Diagnosis delayed but not denied - Sheehan’s syndrome. JNMA J Nepal Med Assoc. 2015;53:31–3. 26983045

[3] Lim CH Han JH Jin J. Electrolyte imbalance in patients with Sheehan’s syndrome. Endocrinol Metab (Seoul). 2015;30:502–8. 26485467 10.3803/EnM.2015.30.4.502 PMC4722405

[4] Kumar N Singh P Kumar J. Recurrent hypoglycaemia: a delayed presentation of Sheehan syndrome. BMJ Case Rep. 2014;2014:bcr2013200991. 10.1136/bcr-2013-200991 PMC4039796 24842349

[5] Laway BA Mir SA Bashir MI. Prevalence of hematological abnormalities in patients with Sheehan’s syndrome: response to replacement of glucocorticoids and thyroxine. Pituitary. 2011;14:39–43. 20798990 10.1007/s11102-010-0255-2

[6] Gokalp D Tuzcu A Bahceci M. Sheehan’s syndrome as a rare cause of anaemia secondary to hypopituitarism. Ann Hematol. 2009;88:405–10. 18797868 10.1007/s00277-008-0607-4

[7] Sadiq S Chowdhury A. A case of Sheehan syndrome 7 years postpartum with transaminitis and hyperlipidemia. Am J Case Rep. 2021;22:e930908. 33951030 10.12659/AJCR.930908 PMC8112285

[8] Jaramillo AM Gonzalez R. Psychiatric and neurocognitive manifestations of Sheehan syndrome: a case report. Prim Care Companion CNS Disord. 2017;19:1. 10.4088/PCC.16l01996 28234440

[9] Karaca Z Laway BA Dokmetas HS. Sheehan syndrome. Nat Rev Dis Primers. 2016;2:16092. 28004764 10.1038/nrdp.2016.92

[10] Matsuwaki T Khan KN Inoue T. Evaluation of obstetrical factors related to Sheehan syndrome. J Obstet Gynaecol Res. 2014;40:46–52. 23945005 10.1111/jog.12119

[11] Dinc H Esen F Demirci A. Pituitary dimensions and volume measurements in pregnancy and post partum MR assessment. Acta Radiol. 1998;39:64–9. 9498873 10.1080/02841859809172152

[12] Scheithauer BW Sano T Kovacs KT. The pituitary gland in pregnancy: a clinicopathologic and immunohistochemical study of 69 cases. Mayo Clin Proc. 1990;65:461–74. 2159093 10.1016/s0025-6196(12)60946-x

[13] Dejager S Gerber S Foubert L. Sheehan’s syndrome: differential diagnosis in the acute phase. J Intern Med. 1998;244:261–6. 9747750 10.1046/j.1365-2796.1998.00370.x

[14] Ramiandrasoa C Castinetti F Raingeard I. Delayed diagnosis of Sheehan’s syndrome in a developed country: a retrospective cohort study. Eur J Endocrinol. 2013;169:431–8. 23864341 10.1530/EJE-13-0279

[15] Ozkan Y Colak R. Sheehan syndrome: clinical and laboratory evaluation of 20 cases. Neuro Endocrinol Lett. 2005;26:257–60. 15990732

[16] Laway BA Mir SA Gojwari T. Selective preservation of anterior pituitary functions in patients with Sheehan’s syndrome. Indian J Endocrinol Metab. 2011;15(Suppl 3):S238–41. 22029030 10.4103/2230-8210.84874 PMC3183521

[17] Diri H Tanriverdi F Karaca Z. Extensive investigation of 114 patients with Sheehan’s syndrome: a continuing disorder. Eur J Endocrinol. 2014;171:311–8. 24917653 10.1530/EJE-14-0244

[18] De Bellis A Kelestimur F Sinisi AA. Anti-hypothalamus and anti-pituitary antibodies may contribute to perpetuate the hypopituitarism in patients with Sheehan’s syndrome. Eur J Endocrinol. 2008;158:147–52. 18230820 10.1530/EJE-07-0647

[19] Du GL Liu ZH Chen M. Sheehan’s syndrome in Xinjiang: clinical characteristics and laboratory evaluation of 97 patients. Hormones (Athens). 2015;14:660–7. 26732159 10.14310/horm.2002.1624

